# Association Between Pyrethroid Exposure Levels and Obesity/Cardiovascular Indicators in Korean Adults: Focused on the 2nd National Environmental Health Survey (2012–2014)

**DOI:** 10.3390/toxics13110958

**Published:** 2025-11-05

**Authors:** Eunbee Bang, Youngwook Lim, Kiyoun Kim

**Affiliations:** 1 Graduate School of Safety Engineering, Seoul National University of Science and Technology, Seoul 01811, Republic of Korea; ebhack92@naver.com; 2 Institute for Environmental Research, Yonsei University, Seoul 03722, Republic of Korea; 3 Department of Safety Engineering, Seoul National University of Science and Technology, Seoul 01811, Republic of Korea

**Keywords:** pyrethroids, 3-PBA, obesity, cardiovascular disease, national environmental health survey

## Abstract

**Background/Objectives**: This study evaluated associations between urinary 3-phenoxybenzoic acid (3-PBA), a metabolite of pyrethroids, and cardiometabolic indicators in a nationally representative sample of Korean adults using data from the 2nd Korean National Environmental Health Survey (KoNEHS 2012–2014). **Methods**: Urinary 3-PBA concentrations were creatinine-adjusted; participants with urinary creatinine < 0.3 or > 3.0 g/L were excluded. Associations with triglycerides, BMI, HDL cholesterol, TSH, and T4 were analyzed using non-parametric tests and multiple regression, with additional verification through log-transformed variables and multiple-comparison control. **Results**: Urinary 3-PBA levels were higher in females, increased with age, and were elevated among rural residents and frequent pesticide users. Triglycerides and TSH showed positive associations with 3-PBA, whereas T4 showed a negative association. BMI displayed a weak negative correlation without consistent significance, and HDL cholesterol was not statistically significant. In multiple regression models, triglycerides, TSH, and T4 remained significantly associated with urinary 3-PBA. **Conclusions**: Statistically significant associations were observed between urinary 3-PBA concentrations and several cardiometabolic indicators, including triglycerides, TSH, and T4, in Korean adults. These findings suggest that even low-level environmental exposure to pyrethroids may influence lipid metabolism and thyroid function. Given the cross-sectional design and the short biological half-life of 3-PBA, the results should be interpreted as associations rather than causation, highlighting the need for longitudinal studies and continued biomonitoring.

## 1. Introduction

According to the U.S. Environmental Protection Agency (EPA), global pesticide usage reached approximately 5.2 billion pounds in 2007—a 700% increase since the 1960s [1]. In South Korea, pesticide use has increased almost 20-fold during the past five decades across both agricultural and urban regions [2]. This global trend reflects the widespread and growing use of pesticides, including insecticides, over recent decades [3].

Pyrethroid insecticides are synthetic compounds derived from natural pyrethrins and are widely used for household and agricultural pest control. Common examples include cypermethrin, permethrin, and deltamethrin. These compounds typically consist of acids and alcohols. Pyrethroids exert their effects by binding to sodium channels in the nerve membranes, inducing abnormal action potentials and excessive neural stimulation, leading to convulsions and paralysis in insects [4].

According to the World Health Organization (WHO), pyrethroids are minimally absorbed through the skin and are primarily absorbed through oral ingestion and inhalation. High-dose oral intake can result in central nervous system abnormalities, such as excitation and seizures. While mammals, including humans, possess hydrolytic enzymes (esterases) capable of metabolizing pyrethroids, insects and fish lack such enzymes, making pyrethroids more toxic to these species. In mammals, most pyrethroids are hydrolyzed and metabolized. Metabolites of pyrethroids include 3-PBA, cis-DBCA, cis- and trans-DCCA, and F-FBA. Among them, 3-Phenoxybenzoic acid (3-PBA) is a common metabolite of cypermethrin, deltamethrin, permethrin, and cyfluthrin, making it a useful biomarker for internal exposure. In the human body, pyrethroids are hydrolyzed into 3-phenoxybenzyl alcohol and are ultimately oxidized to 3-PBA, which is excreted in urine.

Due to their relatively lower toxicity compared to other pesticides such as organophosphates and carbamates, pyrethroids have been widely used since the 1980s [5,6]. Currently, they account for over 30% of the global pesticide market and are the most commonly used insecticides for indoor pest control [7].

Although pyrethroids primarily act on the insect nervous system, chronic human exposure has been associated with hearing loss, endocrine disruption, and hormonal imbalance [8,9,10].

Numerous animal and epidemiological studies have shown that exposure to pyrethroid insecticides disrupts glucose and lipid metabolism and may increase the risk of metabolic disorders such as diabetes. For example, chronic exposure to allethrin and prallethrin has been reported to alter plasma biochemical profiles [11,12]. In another study, Xiao et al. (2017) [13] found that female mice exposed to permethrin alongside a high-fat diet exhibited increased insulin resistance and altered glucose metabolism. Additional studies have demonstrated that mice exposed to cypermethrin and deltamethrin showed elevated blood glucose levels compared to unexposed controls. These effects are thought to result from changes in the activity of key regulators of adipocyte differentiation and insulin resistance, as well as oxidative stress-induced pancreatic β-cell dysfunction [14].

Thus, long-term exposure to pyrethroids may interfere with lipid metabolism and glucose regulation, potentially leading to diabetes and other metabolic disorders. Epidemiological studies have also suggested associations between occupational exposure to pyrethroids and metabolic diseases or diabetes. For example, Hansen et al. (2014) [15] found that pesticide sprayers had a higher prevalence of prediabetes than controls. Similarly, Wang et al. (2011) [16] reported abnormal glucose regulation and observed cases of diabetes among workers exposed to pyrethroids at a pesticide manufacturing facility in China.

Globally, urinary 3-phenoxybenzoic acid (3-PBA) has been widely detected in human biomonitoring studies, with detection frequencies exceeding 80% in several countries [17,18]. These findings indicate that human exposure to pyrethroids is pervasive and underscore the importance of understanding their potential metabolic and endocrine effects at the population level.

In Korea, Choi and Moon (2022) reported a significant association between urinary 3-PBA and self-reported diabetes in adults [19]. However, comprehensive studies examining pyrethroid exposure and cardiometabolic indicators such as obesity, triglycerides, and thyroid hormones in the general Korean population remain limited.

Therefore, this study aimed to investigate urinary 3-PBA levels and examine their associations with cardiometabolic indicators—including BMI, triglycerides, HDL cholesterol, TSH, and T4—using data from the 2nd Korean National Environmental Health Survey (KoNEHS 2012–2014). The study also acknowledges the cross-sectional design and the short half-life of 3-PBA, interpreting the findings as associations rather than causation. This study provides a foundation for future research on the endocrine-disrupting potential of pyrethroids.

## 2. Materials and Methods

### 2.1. Study Population

This study utilized data from the 2nd Korean National Environmental Health Survey (KoNEHS, 2012–2014), provided by the Ministry of Environment, which included data on urinary 3-phenoxybenzoic acid (3-PBA) concentrations as well as demographic variables such as sex, age, body weight, and lifestyle habits. KoNEHS has been conducted every three years since 2009 by the Ministry of Environment to monitor environmental exposures in the general adult population of South Korea. It provides information on internal exposure levels to environmental hazardous substances and associated sociodemographic and behavioral characteristics in the Korean population.

Samples in KoNEHS were selected using a two-stage stratified sampling design stratified by sex, age, and region, ensuring national representativeness. The survey protocol was approved by the Institutional Review Board (IRB) of the National Institute of Environmental Research (NIER).

The 2nd cycle survey included clinical examinations and questionnaires administered to adults aged 19 years and older in 400 enumeration districts over a period of three years. As this study involved urinary concentrations, creatinine correction was applied, and values with urinary creatinine concentrations outside the range of 0.3–3 g/L were excluded for data accuracy. Consequently, from 6478 participants who completed both the questionnaire and biospecimen collection, a final sample of 5493 individuals was selected for analysis after excluding missing values and applying creatinine correction.

### 2.2. Study Methods

#### 2.2.1. Data Collection

All data were drawn from the raw clinical and survey data of the 2nd Korean National Environmental Health Survey (KoNEHS, 2012–2014). The KoNEHS was conducted with approval from the Institutional Review Board of the National Institute of Environmental Research (NIER; Approval No. Environmental Health Research Division-1805, approved on 27 July 2012). Written informed consent was obtained from all participants prior to data collection.

To assess pyrethroid exposure and metabolic indicators, the following variables were collected: urinary 3-PBA and creatinine; serum triglycerides, total cholesterol, and HDL cholesterol (analyzed using automated enzymatic assays, reference ranges: TG 35–150 mg/dL, HDL 40–60 mg/dL); BMI; thyroid hormones (TSH and T4, measured by chemiluminescent immunoassay, reference ranges: TSH 0.35–4.94 μIU/mL, T4 0.8–1.7 ng/dL); smoking and alcohol frequency; and insecticide/pesticide use frequency. Due to the absence of LDL cholesterol values, HDL cholesterol was used instead to examine potential negative correlations with 3-PBA.

BMI was calculated based on height and weight measurements. Insecticide use was assessed by averaging the frequency of usage of insect-control products (e.g., mothballs, chemical agents, mosquito repellents) across seasons, while pesticide use was derived from the seasonal average usage of pesticides for pest control.

#### 2.2.2. Measurement of Urinary 3-PBA Concentration

Urine samples were collected on-site and immediately stored at 0–4 °C before being transported to the laboratory. Samples were frozen at –20 °C within 24 h. Urine samples were pretreated using a liquid–liquid extraction method and analyzed via gas chromatography–mass spectrometry (GC-MS; Clarus 600T, Perkin-Elmer, Waltham, MA, USA). Detailed analytical methods for 3-PBA are available in the KoNEHS Phase 2 Manual for the Analysis of Environmental Hazardous Substances in Biological Samples. Internal quality control followed the guidelines of the National Institute of Environmental Research (NIER). External quality control was conducted by all participating institutions through the Korean Occupational Safety and Health Agency’s special health checkup quality control program and the German G-EQUAS (External Quality Assessment Scheme for Analysis of Organic Chemicals in Biological Materials), both of which were successfully certified. The limit of detection (LOD) for 3-PBA was 0.15 μg/L. Values below the LOD were replaced with LOD/√2 (0.11 μg/L).

### 2.3. Statistical Analysis

All statistical analyses were performed using SAS version 9.4. Because urinary 3-PBA and several biochemical indicators exhibited right-skewed distributions, log-transformed variables (ln [3-PBA], ln[triglycerides], ln[TSH]) were also analyzed. Group differences were tested using the Wilcoxon rank-sum and the Kruskal–Wallis tests, followed by Bonferroni post hoc correction. Differences in urinary 3-PBA levels were also examined according to residential area and pesticide use frequency. Correlations between urinary 3-PBA and triglycerides, HDL cholesterol, BMI, TSH, and T4 were assessed using Spearman correlation coefficients. Multiple linear regression models were applied to examine significant associations while adjusting for age, sex, residence, alcohol consumption, and smoking. Logistic regression was further conducted to estimate odds ratios (ORs) and 95% confidence intervals (CIs) for obesity (BMI ≥ 25 kg/m^2^). *p*-values were adjusted for multiple comparisons using the Bonferroni correction.

## 3. Results

A total of 5493 adults aged ≥19 years from the 2nd Korean National Environmental Health Survey (KoNEHS 2012–2014) were included in the final analysis after applying the creatinine exclusion criteria (<0.3 or >3.0 g/L). The study population consisted of 2542 men and 2951 women.

Participants were distributed as follows: 471 aged 19–29, 913 aged 30–39, 1056 aged 40–49, 1230 aged 50–59, 1107 aged 60–69, and 716 aged ≥70 years.

The mean age was 51.6 years, and the average height and weight were 161.78 cm and 63.78 kg, respectively.

Females exhibited significantly higher creatinine-adjusted urinary 3-PBA levels than males (median 4.7 μg/g vs. 3.4 μg/g, *p* < 0.001; Table 1).

Secondly, non-parametric Kruskal–Wallis tests were conducted to analyze differences in creatinine-adjusted urinary 3-PBA concentrations across age groups.

To minimize potential confounding by residential area, the analysis was stratified into urban and rural subgroups.

Among the 4334 urban adults, 3-PBA concentrations increased progressively with age (Table 2), ranging from 1.70 ± 3.71 μg/g in the 19–29 year group to 5.78 ± 13.20 μg/g in those aged ≥ 70 years.

This age-related trend was statistically significant (*p* < 0.001; the Kruskal–Wallis test), confirming that 3-PBA exposure tends to rise with advancing age in the urban population.

Next, urinary 3-PBA concentrations were analyzed by age group among 411 rural adults. Similarly to the urban subgroup, creatinine-adjusted 3-PBA levels showed an increasing trend with age, from 2.45 ± 1.91 μg/g in participants aged 19–29 to 7.26 ± 13.56 μg/g in those aged ≥ 70 years.

Although the overall Kruskal–Wallis test suggested an upward trend (*p* < 0.001; Table 3), the Bonferroni-adjusted pairwise comparisons did not remain statistically significant, likely due to the limited sample size in the rural subgroup (*n* = 411).

Thirdly, urinary 3-PBA concentrations were compared by residential location (urban vs. rural). Because 3-PBA levels tended to increase with age in both regions, the analysis controlled for this effect by stratifying participants into two age groups: 19–49 years and ≥50 years. Among 2152 adults aged 19–49 years, participants living in rural areas had significantly higher creatinine-adjusted urinary 3-PBA levels than those living in urban areas (mean ± SD: 3.29 ± 3.73 vs. 2.39 ± 3.18 μg/g; *p* < 0.001, Wilcoxon rank–sum test; Table 4). A similar trend was observed among 2593 adults aged ≥ 50 years (5.87 ± 9.34 vs. 5.17 ± 10.64 μg/g; *p* = 0.060, Wilcoxon rank-sum test; Table 5), though the difference did not reach statistical significance.

Fourth, non-parametric Kruskal–Wallis tests were conducted to evaluate differences in creatinine-adjusted urinary 3-PBA concentrations according to the frequency of insecticide and pesticide use.

For both insecticides and pesticides, higher usage frequency was significantly associated with higher 3-PBA levels (*p* < 0.001; Kruskal–Wallis test; Table 6 and Table 7).

Specifically, mean 3-PBA concentrations increased from 2.37 μg/g among non-users to 4.22 μg/g among almost-daily insecticide users, and from 3.58 μg/g to 4.91 μg/g with increasing pesticide use frequency.

These findings indicate a clear dose–response relationship between chemical-use frequency and internal pyrethroid exposure.

Next, to examine the correlations between creatinine-adjusted urinary 3-PBA concentrations and obesity- and cardiovascular-related indicators, relevant variables from the 2nd Korean National Environmental Health Survey (KoNEHS 2012–2014) were selected based on previous epidemiological studies.

The indicators included alcohol consumption, smoking, sex, triglycerides, HDL cholesterol, BMI, total lipids, and thyroid hormones (TSH and T4).

Among these, alcohol consumption, smoking, and sex were categorical variables and were therefore excluded from the correlation analysis.

Spearman’s rank correlation tests were performed between urinary 3-PBA and continuous variables. The results showed the following:

- Triglycerides, BMI, and TSH exhibited statistically significant correlations with urinary 3-PBA (*p* < 0.05);
- T4 showed a significant negative correlation;
- HDL cholesterol and total lipids were not significantly correlated (*p* > 0.05; Table 8).
- Specifically, BMI displayed a weak negative correlation (r = –0.015, *p* < 0.001; Spearman correlation), confirming the directionality observed in Table 8.

Finally, multiple linear regression analyses were performed to examine the associations between creatinine-adjusted urinary 3-PBA concentrations and the obesity- and cardiovascular-related indicators that had previously shown significant correlations.

Among the variables tested in the correlation analysis—triglycerides, HDL cholesterol, BMI, total lipids, TSH, and T4—those without statistically significant correlations (HDL and total lipids) were excluded.

The remaining four indicators (triglycerides, BMI, TSH, and T4) were used as dependent variables, and separate regression models were constructed for each.

For the models with triglycerides and BMI as dependent variables, urinary 3-PBA concentration was entered as the main independent variable.

For the thyroid hormone models (TSH and T4), both hormones were included simultaneously as independent variables to account for their physiological interrelationship.

All models were adjusted for age, sex, residential area, alcohol consumption, and smoking as covariates.

The regression analyses revealed that urinary 3-PBA was

- positively associated with triglycerides (β = 0.540, *p* = 0.031; Table 9);
- not significantly associated with BMI (β = –0.006, *p* = 0.35; Table 10).
- modestly but significantly associated with TSH (β = –0.015, *p* = 0.034; Table 11);
- negatively associated with T4 (β = –0.009, *p* = 0.002; Table 12).

Because several variables exhibited right-skewed distributions, analyses using log-transformed variables (ln[3-PBA], ln[triglycerides], ln[TSH]) were additionally conducted and produced consistent results.

All *p*-values were adjusted for multiple comparisons using the Bonferroni correction to control the family-wise Type I error rate.

## 4. Discussion

This study utilized data from the 2nd Korean National Environmental Health Survey (2012–2014), representative of the general Korean adult population, to investigate the associations between urinary 3-phenoxybenzoic acid (3-PBA)—a non-specific metabolite of pyrethroid insecticides—and indicators related to obesity and cardiovascular health.

Previous studies have suggested that pyrethroid exposure may contribute to metabolic disorders such as obesity and diabetes through endocrine-disrupting pathways that also influence cardiovascular health. Leso et al. (2017) [20] observed an increased prevalence of diabetes among workers occupationally exposed to pyrethroids, suggesting interference with glucose homeostasis. Other reports have proposed that endocrine disruption may alter adipogenesis and lipid metabolism, underscoring the broader metabolic impact of pyrethroid exposure [21].

By analyzing urinary 3-PBA concentrations as a biomarker of exposure, this study identified exposure levels in Korean adults and examined their relationships with obesity- and cardiovascular-related indicators, thereby providing population-based evidence of possible endocrine-disrupting effects.

However, several methodological limitations should be considered.

First, the study used secondary data from a retrospective cross-sectional survey (KoNEHS), which was not designed for this specific hypothesis; thus, causal inference is not possible, and future cohort studies are warranted.

Second, pyrethroids are non-persistent and rapidly metabolized. Upon exposure, they are hydrolyzed to 3-phenoxybenzyl alcohol and oxidized to 3-PBA, which is excreted in urine. Previous research [22] has found a half-life of approximately 1.8 days, with near-complete elimination within four days.

Third, although pyrethroids were once considered to have minimal chronic effects due to rapid hydrolysis, their mechanisms of action on metabolic health remain uncertain.

Current evidence suggests they may (1) affect adipocyte differentiation and insulin resistance, (2) induce oxidative stress that impairs β-cell function, and (3) alter thyroid hormone levels, disrupting lipid and glucose regulation and thereby contributing to metabolic disorders.

Although these mechanisms are not fully elucidated, growing evidence supports their relevance. For example, urinary 3-PBA has been linked with increased BMI in Korean adults using the 1st KoNEHS data [23], consistent with the present findings. Dietary habits also influence urinary 3-PBA concentrations [24], possibly explaining exposure variability. Moreover, recent studies report associations between chronic pyrethroid exposure and metabolic or cardiovascular disturbances in younger populations [25,26].

This study contributes by (1) confirming exposure levels of pyrethroids in a representative Korean sample, (2) demonstrating consistent associations between pyrethroid exposure and indicators of obesity and cardiovascular risk, and (3) suggesting that even low-level, chronic environmental exposure—rather than occupational high-dose exposure—may have measurable effects on adult health.

Addressing these limitations and conducting follow-up studies that incorporate socioeconomic status, dietary habits, and co-exposure to other environmental chemicals could further clarify the observed associations and strengthen the evidence base for public health interventions.

## 5. Conclusions

This study used data from the 2nd Korean National Environmental Health Survey (2012–2014), representative of the Korean adult population, to evaluate urinary 3-phenoxybenzoic acid (3-PBA) exposure levels and examine their associations with obesity- and cardiovascular-related indicators.

The analysis showed statistically significant differences in urinary 3-PBA levels by sex, with higher levels observed in females. Exposure also increased significantly with age and was higher among rural residents than urban residents. Moreover, urinary 3-PBA concentrations were significantly higher among participants who reported more frequent use of insecticides and pesticides, showing a significant positive correlation—more frequent use was associated with higher urinary 3-PBA levels.

Correlation analyses revealed that triglycerides, BMI, and thyroid hormone TSH were positively associated with urinary 3-PBA, whereas HDL cholesterol and thyroid hormone T4 showed negative associations. In multiple regression models adjusted for age, sex, residential area, alcohol consumption, and smoking, significant associations remained for triglycerides, HDL, TSH, and T4, while BMI showed no consistent significance after adjustment.

These associations align with international findings indicating systemic metabolic and cardiovascular effects of low-dose environmental pyrethroid exposure [27,28].

Logistic regression analysis using BMI-based obesity status (BMI ≥ 25 kg/m^2^) as the dependent variable and urinary 3-PBA as the independent variable demonstrated that each unit increase in urinary 3-PBA was associated with a statistically significant increase in the odds of being obese by approximately one unit. Despite the fact that pyrethroid compounds are not bioaccumulative or persistent, these results suggest that chronic low-level environmental exposure—rather than high-dose occupational exposure—may affect obesity and cardiovascular indicators in adults. Therefore, in-depth studies on the chronic effects of pyrethroid exposure are needed to improve public health outcomes.

Although this study was based on secondary cross-sectional data, which precludes causal inference, it contributes meaningful evidence supporting previous findings and highlights the potential influence of pyrethroid exposure on obesity- and cardiovascular-related health outcomes. Continued biomonitoring and longitudinal studies are needed to clarify the long-term health implications of chronic pyrethroid exposure in the general population.

## Figures and Tables

**Table 1:** Urinary 3-PBA Concentrations by Sex (μg/g creatinine).

Group	Number	Mean ± SD	*p*-Value
Total	5493	4.10 ± 8.00	<0.0001 *
Male	2542	3.41 ± 8.98	
Female	2951	4.70 ± 7.16	

* *p* < 0.001 (Wilcoxon rank-sum test).

**Table 2:** Urinary 3-PBA concentrations by age group (Urban) (μg/g creatinine).

Age Group	Number	Mean ± SD	*p*-Value
19–29	398	1.70 ± 3.71	<0.0001 *
30s	771	2.23 ± 2.55	
40s	846	2.85 ± 3.36	
50s	953	4.81 ± 10.37	
60s	817	5.18 ± 8.86	
70+	549	5.78 ± 13.20	

* *p* < 0.001 (Kruskal–Wallis test).

**Table 3:** Urinary 3-PBA concentrations by age group (rural) (μg/g creatinine).

Age Group	Number	Mean ± SD	*p*-Value
19–29	16	2.45 ± 1.91	<0.0001 *
30s	41	2.96 ± 4.05	
40s	80	3.63 ± 3.82	
50s	110	5.39 ± 9.40	
60s	104	5.57 ± 5.55	
70+	60	7.26 ± 13.56	

* *p* < 0.001 (Kruskal–Wallis, Bonferroni corrected).

**Table 4:** Urinary 3-PBA concentrations by area of residence (Age 19–49) (μg/g creatinine).

Residence	Number	Mean ± SD	*p*-Value
Total	2152	2.45 ± 3.22	<0.0001 *
Urban	2015	2.39 ± 3.18	
Rural	137	3.29 ± 3.73	

* *p* < 0.001 (Wilcoxon rank–sum test).

**Table 5:** Urinary 3-PBA concentrations by area of residence (Age 50+) (μg/g creatinine).

Residence	Number	Mean ± SD	*p*-Value
Total	2593	5.25 ± 10.50	<0.0597
Urban	2319	5.17 ± 10.64	
Rural	274	5.87 ± 9.34	

**Table 6:** Urinary 3-PBA concentrations by insecticide use frequency (μg/g creatinine).

Usage Frequency	Group No.	Number	Mean	*p*-Value
None	1	349	2.37	<0.0001 *
2–3 times/month or less	2	550	3.15	
Almost daily	3	1437	4.22	

* *p* < 0.001 (Kruskal–Wallis test).

**Table 7:** Urinary 3-PBA concentrations by pesticide use frequency (μg/g creatinine).

Usage Frequency	Group No.	Number	Mean	*p*-Value
None	1	1937	3.58	<0.0001 *
2–3 times/month or less	2	492	3.35	
Almost daily	3	483	4.91	

* *p* < 0.001 (Kruskal–Wallis test).

**Table 8:** Correlation between Urinary 3-PBA concentration and obesity/cardiovascular indicators.

Indicator	Correlation Coefficient^®^	*p*-Value
Triglycerides	0.036	0.008 *
HDL	0.008	0.407
BMI	−0.015	<0.0001 **
Total Lipids	0.065	0.022
TSH	0.031	<0.0001 **
T4	−0.050	0.0002 **

* *p* < 0.01; ** *p* < 0.001 (Spearman correlation).

**Table 9:** Regression analysis for triglyceride level by independent variables (3-PBA).

Variable	Beta	SE	*p*-Value	VIF	Pr > F
Gender (M = 1, F = 2)	−25.274	4.419	<0.0001 ***	1.302	<0.0001 *
Age	0.808	0.135	<0.0001 ***	1.122	
Residence (Urban = 1, Rural = 2)	6.983	6.886	0.311	1.008	
Alcohol	21.378	2.727	<0.0001 ***	1.250	
Smoking	2.304	2.128	0.279	1.159	
Urinary 3-PBA	0.540	0.250	0.031 *	1.042	

Adjusted by gender, age, alcohol, and smoking; * *p* < 0.05; *** *p* < 0.001 (multiple linear regression)

**Table 10:** Regression analysis for BMI by independent variables (3-PBA).

Variable	Beta	SE	*p*-Value	VIF	Pr > F
Gender (M = 1, F = 2)	−0.543	0.112	<0.0001 ***	1.302	<0.0001 *
Age	0.026	0.003	<0.0001 ***	1.120	
Residence (Urban = 1, Rural = 2)	0.422	0.174	0.016 *	1.008	
Alcohol	−0.031	0.069	0.653	1.248	
Smoking	0.011	0.054	0.832	1.159	
Urinary 3-PBA	−0.006	0.006	0.348	1.040	

Adjusted by gender, age, alcohol, and smoking; * *p* < 0.05; *** *p* < 0.001 (multiple linear regression).

**Table 11:** Regression analysis for TSH level by independent variables (3-PBA).

Variable	Beta	SE	*p*-Value	VIF	Pr > F
Gender (M = 1, F = 2)	0.264	0.126	0.036 *	1.301	<0.0001 *
Age	0.010	0.004	0.008 **	1.122	
Residence (Urban = 1, Rural = 2)	−0.186	0.196	0.344	1.008	
Alcohol	−0.198	0.078	0.011 *	1.253	
Smoking	−0.125	0.061	0.041 *	1.171	
T4	−0.505	0.036	<0.0001 ***	1.015	
Urinary 3-PBA	−0.015	0.007	0.034 *	1.043	

Adjusted by gender, age, alcohol, and smoking; * *p* < 0.05; ** *p* < 0.01; *** *p* < 0.001 (multiple linear regression).

**Table 12:** Regression analysis for T4 level by independent variables (3-PBA).

Variable	Beta	SE	*p*-Value	VIF	Pr > F
Gender (M = 1, F = 2)	0.059	0.050	0.244	1.302	<0.0001 *
Age	−0.001	0.002	0.556	1.124	
Residence (Urban = 1, Rural = 2)	0.057	0.078	0.468	1.008	
Alcohol	0.110	0.031	0.0004 ***	1.252	
Smoking	−0.176	0.024	<0.0001 ***	1.159	
TSH	−0.080	0.006	<0.0001 ***	1.009	
Urinary 3-PBA	−0.009	0.003	0.002 **	1.042	

Adjusted by gender, age, alcohol, and smoking; * *p* < 0.05; ** *p* < 0.01; *** *p* < 0.001 (multiple linear regression).

## Data Availability

The data that support the findings of this study are available from the National Institute of Environmental Research, Ministry of Environment, Korea (Project Code: 2014-01-01-074), but restrictions apply to the availability of these data, which were used under license for the current study, and so are not publicly available. Data are, however, available from the authors upon reasonable request and with permission from the NIER.
